# Investigation of Growth Factor and Tenocyte Proliferation Induced by Platelet Rich Plasma (PRP) in a 3-Chamber Co-Culture Device

**DOI:** 10.3390/mi9090446

**Published:** 2018-09-07

**Authors:** Chih-Hao Chiu, Rei Higashikawa, Wen-Ling Yeh, Kin Fong Lei, Alvin Chao-Yu Chen

**Affiliations:** 1 Department of Orthopedic Surgery, Chang Gung Memorial Hospital, Taoyuan 333, Taiwan; joechiu0115@gmail.com (C.-H.C.); phoebeling225@gmail.com (R.H.); 2 Bone and Joint Research Center, Chang Gung Memorial Hospital, Linkou 333, Taiwan; yeh610128@gmail.com; 3 Department of Orthopedic Surgery, Chang Gung Memorial Hospital, Linkou 333, Taiwan; 4 Graduate Institute of Biomedical Engineering, Chang Gung University, Taoyuan 333, Taiwan; 5 Department of Radiation Oncology, Chang Gung Memorial Hospital, Linkou 333, Taiwan

**Keywords:** platelet-rich plasma, PRP, tenocytes, co-culture, microdevices

## Abstract

The platelet-rich plasma (PRP) has become an attractive topic for soft tissue healing therapy recently. While some clinical reports revealed the effective treatments for knee osteoarthritis, lateral epicondylitis, and rotator cuff tears, other case studies showed that there was no statistically significant healing improvement. The efficacy of the PRP therapy is still unclear clinically. Thus, a significant amount of basic studies should be conducted to optimize the preparation procedure and the platelet concentration of the PRP. In this work, a 3-chamber co-culture device was developed for the PRP study in order to reduce the usage of primary cells and to avoid the PRP gelation effect. The device was a culture, well partitioning into 3 sub-chambers. Tenocytes and PRP could be respectively loaded into the sub-chambers and co-cultured under the interlinked medium. The results showed that a higher platelet number in the PRP could diffuse higher concentration of the growth factors in the medium and induce higher tenocyte proliferation. The 3-chamber co-culture device provides a simple and practical tool for the PRP study. It is potentially applied for optimizing the preparation procedure and platelet concentration of the PRP therapy.

## 1. Introduction

In the research of sports medicine, the use of platelet-rich plasma (PRP) has recently become an attractive approach for stimulating and accelerating the soft tissue healing process [1,2]. The PRP is prepared by centrifuging whole blood drawn from a patient. This centrifugation process can concentrate the platelets and remove the blood cells. As the platelets contain hundreds of growth factors, which are very important in the healing process, the PRP with many more platelets than whole blood may encourage the healing response. Currently, there is no strict definition and preparation procedure of the PRP. Generally, as long as the plasma has a greater concentration of platelets than whole blood, this plasma is called PRP. In a clinical scenario, the PRP with 3–5 times platelet concentration can be used for the tissue injection where healing is desired.

In the past decade, a number of in vitro studies reported that the PRP enhances the proliferation of human muscle, bone, and tendon cells [3,4,5,6,7]. As high concentration of platelet-derived growth factors are generated that mimic physiologic healing, the tissue cells can be stimulated for the repairing process. Moreover, some clinical reports showed effective treatments for knee osteoarthritis, lateral epicondylitis, and rotator cuff tears [8,9,10]. In contrast, other case studies revealed that there was no statistically significant difference in the PRP group and the control group [11,12,13,14,15]. Nowadays, the efficacy of the PRP therapy is still unclear and remains controversial among medical experts. The preparation procedure and the platelet concentration of PRP are still required to conduct significant amount of basic studies [16]. 

Cell culture is a basic bio-technology conducted in the laboratory for studying cellular responses under tested conditions. In the previous studies of the PRP, the cells isolated from the patient’s tissue were cultured on multi-well culture plates, e.g., 12-well and six-well plates [4,5,6]. The PRP derived from whole blood was directly applied to the plates and cell proliferation was investigated under the stimulation of the PRP. The reason for using large culture plates was to avoid gelation of the culture medium. The gelation effect is a major problem in the in vitro studies [6]. The PRP can induce gelation of the entire culture medium when using small culture plates, e.g., 96-well and 24-well plates [17]. Thus, the results may not reasonably reveal the efficacy of the platelet-derived growth factors. Large culture plates, e.g., 12-well and six-well plates were commonly used for the in vitro studies of the PRP. However, because primary cells are isolated from the patient’s tissue, the cell number is limited for each isolation. Additionally, multiple passages result in changes of gene expression of the cells [18,19,20]. This poses a problem in clinical research because freshly isolated cells are not manifold available in a sufficient amounts. 

In order to reduce the usage of primary cells and avoid the PRP gelation effect, a 3-chamber co-culture device was developed in this work. Due to the mature development of the microfabrication technology in the past decades, a lot of microfluidic devices and systems have been developed for various specific applications such as clinical diagnostics and cell-based assays [21,22,23]. Researchers focus on the development of simple and practical devices to solve specific clinical problems. In the current study, the 3-chamber co-culture device was designed for the study of the PRP. The device was composed of 3 sub-chambers separated by a barrier; such that the PRP gel and cells were respectively seeded in different sub-chambers and then cultured with the common culture medium supplied. The cells were stimulated by the platelet-derived growth factors diffusing in the medium. In this work, the PRP generated from human whole blood was prepared by different procedures for centrifuging the PRP in different platelet numbers. Human fibroblast growth factor (FGF), derived from the PRP, was studied in the 3-chamber co-culture device. The results revealed that the platelet numbers in the PRP was proportional to the human FGF concentration. Moreover, tenocytes were isolated from human rotator cuff tendons and co-cultured with the PRP up to seven days. The results showed that the tenocyte proliferation was proportional to the platelet number after the seven-day culture. In summary, the 3-chamber co-culture device is developed in order to reduce the usage of primary cells and avoid the PRP gelation effect. It is simple and practical for the PRP study and allows to large volume production for the in vitro studies using clinical samples.

## 2. Materials and Methods

### 2.1. Preparation of the PRP from Human Whole Blood

**Figure 1:**
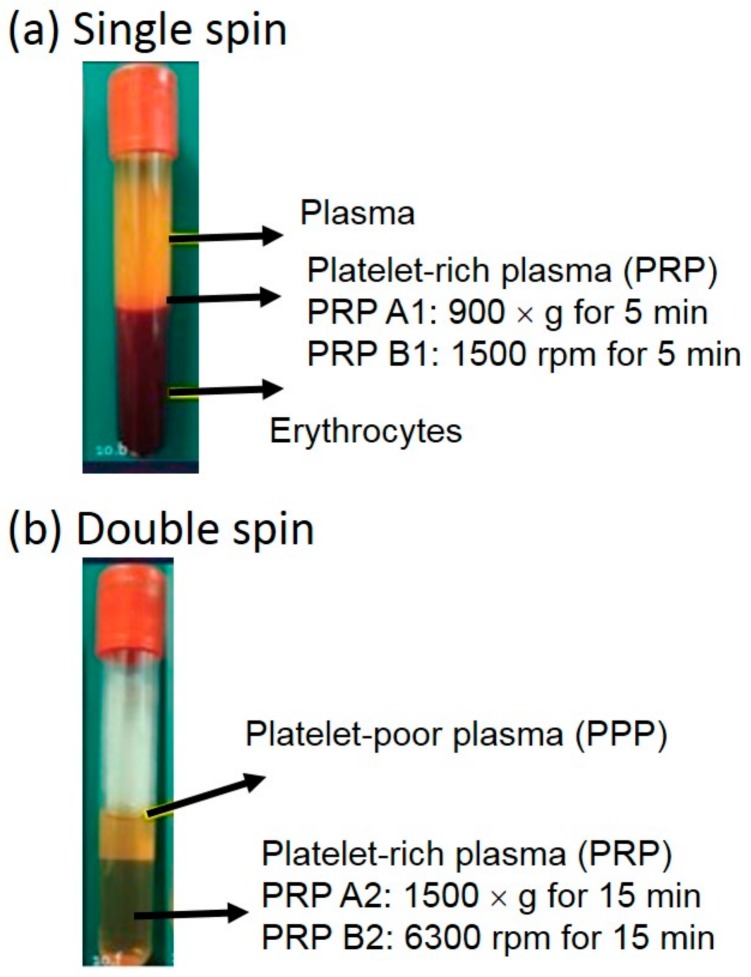
Photograph of the platelet-rich plasma (PRP). (**a**) The PRP A1 and B1 were prepared by collecting the bottom layer of the supernatant after the centrifugation of the whole blood at 900× *g* and 1500 rpm for 5 min, respectively. (**b**) The PRP A2 and B2 were obtained by a condensation centrifugation of 1500× *g* and 6300 rpm for 15 min from the PRP A1 and B1, respectively.

The approval of the collection of human blood samples and tendon tissues while undergoing arthroscopic rotator cuff repair was provided by the Institutional Review Board, Chang Gung Memorial Hospital, Linkou, Taiwan (IRB No. 2016014923). The 20 mL whole blood was drawn from a patient or volunteer in the hospital. The blood was then collected by BD Vacutainer^®^ blood collection tubes (367525; Becton, Dickinson and Company, Franklin Lakes, NJ, USA). Afterwards, the blood was sent to the laboratory and the PRP was prepared by centrifugation process using a bench-top centrifuge machine (Model: 5430R, Eppendorf, Hamburg, Germany). Four different procedures were adopted for preparing the PRP in different platelet numbers, named PRP A1, PRP A2, PRP B1, and PRP B2. Photographs of the PRP are shown in Figure 1. The PRP A1 and B1 were prepared by collecting the bottom layer of the supernatant after the centrifugation of the whole blood at 900× *g* and 1500 rpm for 5 min, respectively. The PRP A2 and B2 were obtained by a condensation centrifugation of 1500× *g* and 6300 rpm for 15 min from the PRP A1 and B1, respectively. The platelet number of the prepared PRP was counted by an automatic hematology analyzer (Model: XT-1800i, Sysmex Corporation, Kobe, Japan). 

### 2.2. Isolation of Tenocytes Obtained from Human Rotator Cuff Tendons

**Figure 2:**
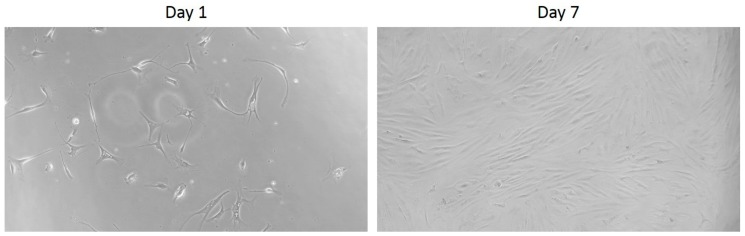
Microscopic images of the tenocytes cultured on day 1 and day 7.

The human rotator cuff tendons were collected during the surgery of arthroscopic rotator cuff repair. Before the surgery, the patient gave informed consent. Tendon tissues were isolated from the edge of torn human rotator cuff tendons. The tenocyte isolation procedure followed the previous protocol with appropriate modifications [24,25]. The tissues were digested in a mixture of enzymatic solution containing 4 mg/mL dispase (Roche, Burgess Hill, UK) and 300 U/mL collagenase Type II (Gibco, Invitrogen, Paisley, UK) at 37 °C for 16 h. Then, the mixture was filtered and centrifuged at 1000 rpm for 5 min at room temperature. The cell pellet was suspended and maintained in culture medium (RPMI 1640 supplemented with 10% FBS and 1% antibiotics) in standard tissue culture flasks. The microscopic images of the tenocytes cultured on day 1 and day 7 are shown in Figure 2. After one passage, the tenocytes were trypsinized using 0.05% trypsin for 3 min, centrifuged at 1200 rpm for 5 min, and resuspended in the culture medium. The tenocytes within 3 passages were used for the further experiments because multiple passages, e.g., over 3 passages, result in changes of gene expression of the cells [18,19,20]. The 1 × 10^4^ cells were seeded in the sub-chamber of the co-culture device for the current study. The cell number was counted before cell seeding using an automated cell counter (Model: Countess II FL; Invitrogen, Carlsbad, CA, USA).

### 2.3. Fabrication of the 3-Chamber Co-Culture Device

**Figure 3:**
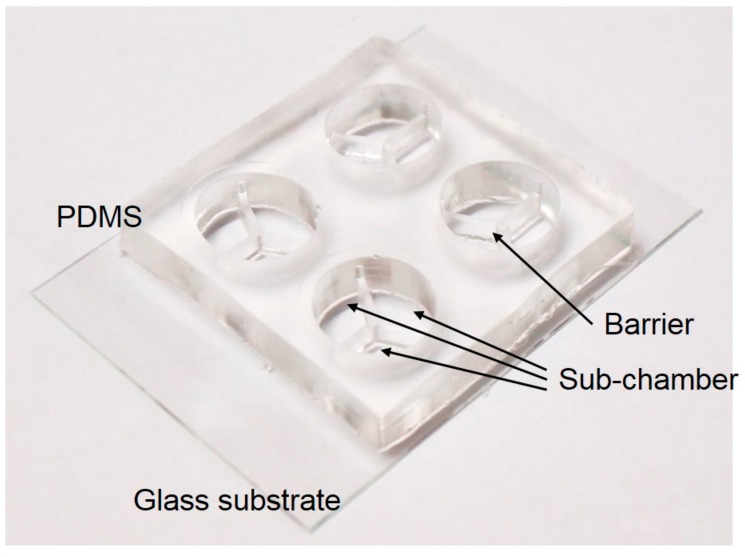
Photograph of the 3-chamber co-culture device.

The 3-chamber co-culture device was developed for the study of the PRP in order to reduce the usage of primary cells and avoid the PRP gelation effect. The device was a circular culture well of 15 mm in diameter and 7 mm in height. It was comprised of 3 sub-chambers separated by a barrier of 3 mm in height and 1 mm in width. A photograph of the 3-chamber co-culture device is shown in Figure 3. The PRP and the tenocytes could be respectively applied to different sub-chambers. After gelation of the PRP and seeding of the tenocytes, a culture medium was added to the culture well and the level of the medium was over the height of the barrier. Therefore, the platelet-derived growth factors diffused in the medium and stimulated the tenocytes. 

The fabrication process of the 3-chamber co-culture device was briefly described. It consisted of a glass substrate and a polydimethylsiloxane (PDMS) (Model: Sylgard^®^ 184; Dow Corning, Midland, MI, USA) layer. The PDMS layer was fabricated by molding from a poly(methacrylate) (PMMA) mold machined by a CNC engraving machine (Model: EGX-400; Roland, Hamamatsu, Japan). The PDMS layer was then bonded to the glass substrate after the treatment of oxygen plasma. Subsequently, the co-culture device was washed by phosphate-buffered saline (PBS; K813-500ML, VWR Life Science, Philadelphia, PA, USA) and kept under an ultraviolet light for further experiments.

### 2.4. Study of the Platelet-Derived Growth Factor

To study the platelet-derived growth factor, 40 μL PRP and 40 μL culture medium were respectively added to one of the sub-chambers of the culture well and kept overnight. After gelation of the PRP, 700 μL culture medium was applied to the culture well. Subsequently, the platelet-derived growth factors diffused in the medium. The supernatant was collected at day 0, 1, 2, and 3 and human FGF was analyzed by using a commercial immunoassay kit (Human FGF basic Quantikine^®^ ELISA kit, R&D Systems, Minneapolis, MN, USA). The human FGF is a growth factor that regulate a broad spectrum of biological functions including cellular proliferation, angiogenesis, wound healing, and tissue repair. Moreover, literature reported that FGF responds for the early stages of tendon healing [26,27,28]. The analytical protocol followed the manufacturer’s instruction. The supernatant was pipetted to a kit supplied microplate that pre-coated with the primary antibody and incubated for 3 h at room temperature. Then, the microplate was washed using the provided buffer. Then, the detection antibody was added and incubated for 1.5 h at room temperature. After washing, the substrate solution was added and incubated for 20 min. The optical density (OD) of the reacted solution was quantified by a microplate reader (Model: ELx800, BioTek Instrument, Inc., Winooski, VT, USA). In addition, a serial dilution of the provided standard FGF solution was utilized to setup the calibration curve. Thus, the actual concentration of the FGF could be calculated based on the calibration curve.

### 2.5. Study of the Tenocyte Proliferation

**Figure 4:**
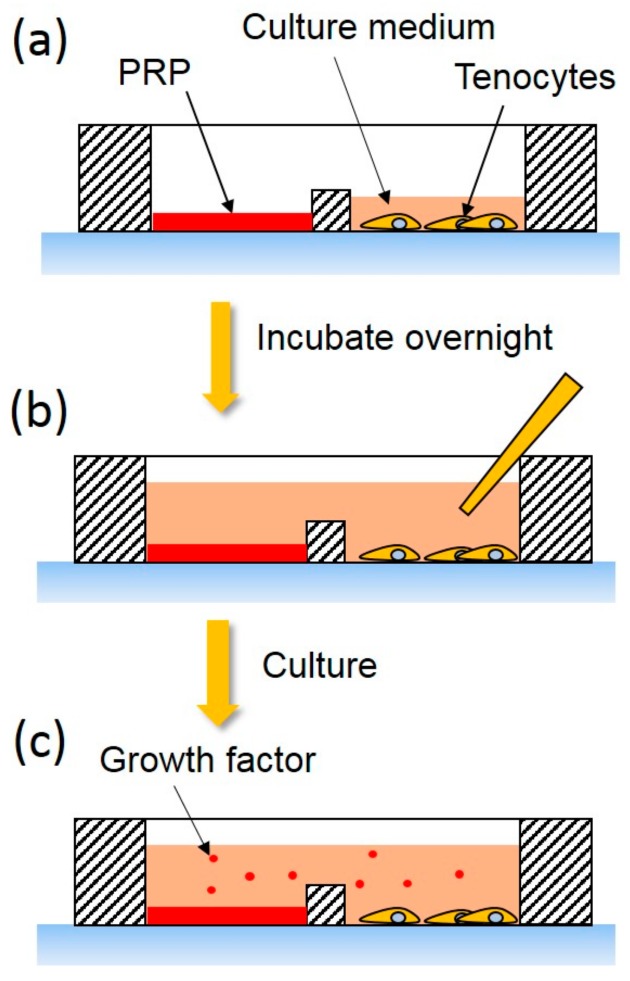
Experimental protocol of the investigation of the proliferation of tenocytes co-culturing with the PRP. (**a**) The PRP and the tenocytes were respectively seeded on the sub-chambers of the device and incubated overnight. (**b**) The culture medium was added to the culture well; therefore, the PRP and the tenocytes were co-cultured in the interlinked medium. (**c**) The platelet-derived growth factors diffused to the medium and affected tenocyte proliferation.

The protocol of this study is illustrated in Figure 4. The 40 μL PRP and 40 μL culture medium were added to one of the sub-chambers of the culture well. The 1 × 10^4^ tenocytes suspended in 100 μL culture medium were respectively seeded on the other 2 sub-chambers. After gelation of the PRP and stabilization of the tenocytes overnight, 500 μL culture medium was applied to the culture well. The platelet-derived growth factors diffused in the medium and stimulated the tenocytes. After a 7-day culture course, a cell number was quantified by the conventional bio-assay, i.e., WST-1 assay (Roche Applied Science, Indianapolis, IN, USA). The culture medium was removed and 100 μL reagent of WST-1 assay in the dilution of 1:10 was respectively added to the sub-chambers. After incubation at 37 °C for 2 h, the supernatant was respectively collected and transferred to a microplate. Color intensity of the reacted product represented by the OD was quantified by the microplate reader. The proliferation ratio was defined as the OD value at the end of the culture course (day 7) and divided by the OD value at day 0.

## 3. Results and Discussion

### 3.1. Study of the PRP Prepared by Different Procedures

**Figure 5:**
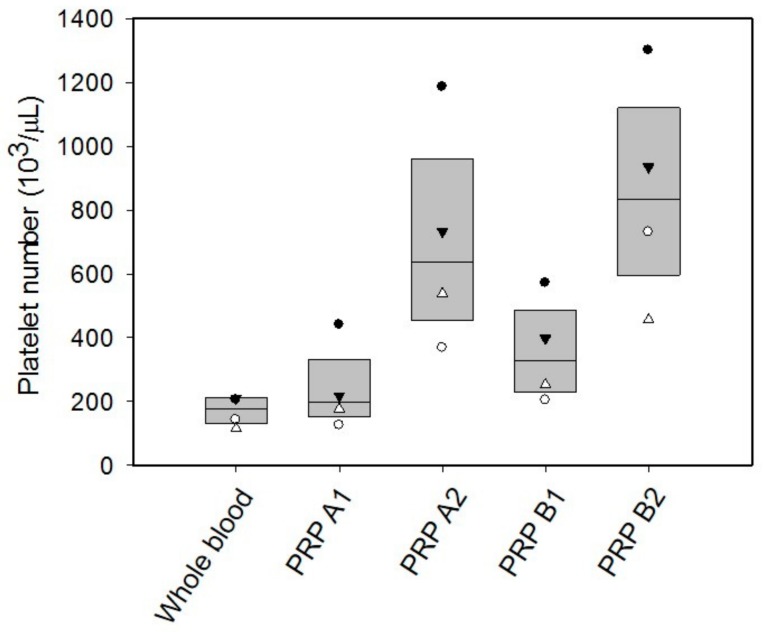
Platelet number in the PRP prepared by 4 different procedures. The blood samples were collected from 4 donors in the ages of 49, 50, 70, and 72. Different symbols represent the data points from different donors. The data are presented as mean ± standard deviation (SD).

**Figure 6:**
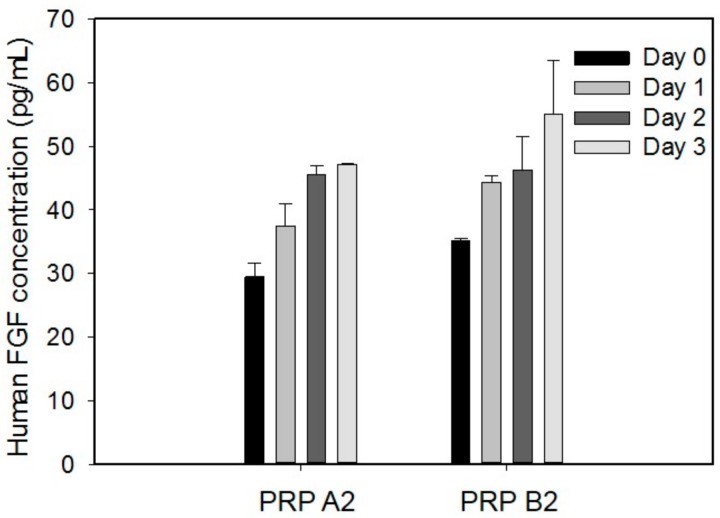
Human FGF (fibroblast growth factor) concentration in the culture medium at day 0, 1, 2, and 3. The PRP A2 and PRP B2 were respectively applied to the culture wells and cultured for 3 days. The supernatant was collected every day and human FGF concentration was measured. The data are the representative results from the blood sample collected from a 70-year donor. The data are collected from 3 repeated experiments and presented as mean ± standard deviation (SD).

**Figure 7:**
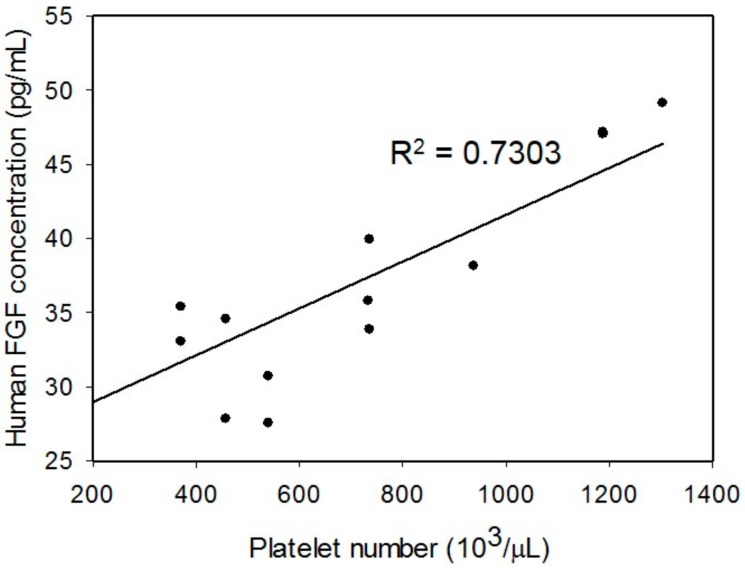
Correlation between the platelet number in the PRP and the human FGF concentration. The blood samples were collected from 4 donors in the ages of 49, 50, 70, and 72. The PRP were respectively prepared by A2 and B2 procedures. Each data point represents the result from 1 experiment.

As the tissue healing process is stimulated by the platelet-derived growth factors, the platelet number of the PRP is expected to have correlation with the concentration of the growth factor. Investigation of the platelet number in the PRP was conducted to quantify the PRP. The blood samples were collected from four donors. The result is shown in Figure 5. Different symbols represent the data points from different donors. The platelet number in the whole blood ranged 150,000 to 200,000 μL^−1^ in our patient or volunteer. That is reasonable as the platelet number in a healthy person is from 150,000 to 400,000 μL^−1^ [29]. Then, the PRP was respectively prepared by four different procedures. Obviously, the platelet number was increased after the centrifugation. The platelet number of the PRP after double spin (including A2 and B2) was higher than those after single spin (including A1 and B1). Therefore, the PRP in a broad range of the platelet number could be prepared by different procedures. Moreover, it is observed that when the whole blood had a lower platelet number, the PRP contained lesser platelets after centrifugation. That indicates the variability of donors. In order to quantify the regenerative capability of the PRP, platelet number in the PRP was used to compensate the effect of donor variability in our study. On the other hand, the platelet-derived growth factor, i.e., FGF, was studied during a three-day culture course. The PRP A2 and PRP B2 were respectively applied to the culture wells and cultured for three days. The supernatant was collected every day and human FGF concentration was measured. Figure 6 shows the representative results from the blood sample collected from a 70-year donor. The result revealed that the FGF concentration was increased with time. That indicated that the PRP required time to release or dissolve the growth factors into the culture medium. In addition, the correlation between the platelet number in the PRP and the human FGF concentration was studied. The blood samples were respectively drawn from four donors. The PRP was respectively prepared by A2 and B2 procedures. The platelet number of the PRP was quantified before the experiments. The FGF concentration was collected and analyzed after culturing the PRP for three-days. The result is shown in Figure 7. It showed that the platelet number had a linear correlation with the FGF concentration with an R-squared value of 0.7303. In addition, the 3-chamber co-culture device was confirmed to be used for the PRP study. 

### 3.2. Proliferation of Human Tenocytes Co-Culturing with the PRP

**Figure 8:**
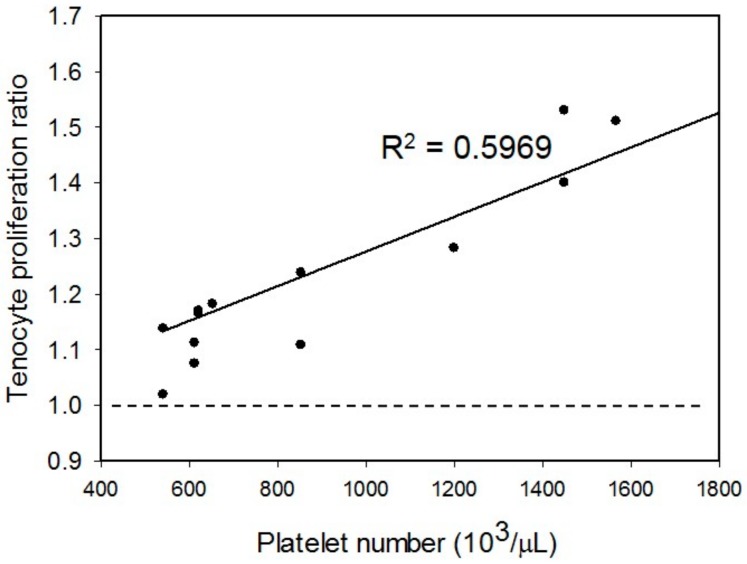
Correlation between the platelet number in the PRP and the tenocyte proliferation ratio. The blood and tissue samples were collected from 8 different patients. The PRP were respectively prepared by A2 and B2 procedures. Each data point represents the result from 1 experiment.

As direct application of the PRP to the culture medium can induce gelation of the entire medium when using small culture plates, e.g., 96-well and 24-well plates, large culture plates, e.g., 12-well and six-well plates, were conventionally used for the in vitro PRP studies to avoid gelation [4,5,6]. Gelation influences the release of growth factors and the effect of cell function. Therefore, the result may not truly reflect the efficacy of the PRP reasonably. Moreover, this gelation situation does not happen in the in vivo environment. Thus, a 3-chamber co-culture device was designed and used for eliminating the gelation effect and reducing the usage of human tenocytes. The PRP was gelled in one of the sub-chambers to avoid the gelation of the entire medium. The correlation between the platelet number in the PRP and the tenocyte proliferation ratio was investigated and the result is shown in Figure 8. The blood and tissue samples were collected from eight different patients. The PRP were respectively prepared by A2 and B2 procedures. The platelet number of the PRP was quantified before the experiments. The tenocytes were co-cultured with the PRP and the tenocyte proliferation ratio was determined after the seven-day culture course. The result confirmed that the tenocyte proliferation could be enhanced when co-culturing with the PRP (all sets of data were higher than one). Moreover, the platelet number in the PRP had a linear correlation with the tenocyte proliferation ratio with an R-squared value of 0.5969. Together with the result from Figure 6, higher platelet number of the PRP could generate a higher concentration of the growth factors in the medium and promote higher tenocyte proliferation ratio. 

Tenocytes are fibroblast-like cells and constitute the cellular component of periarticular tendons. They are responsible for synthesis, production, and maintenance of the extracellular matrix. Additionally, they initiate the regenerative responses following injury or degeneration. However, after the arthroscopic repair of rotator cuff, the re-tear rate has reported to be 25% to 91% [30]. That revealed the tendon-to-bone interface is unsuccessfully reintegrated. That might be because the tenocyte proliferation is very slow especially for people above the age of 65 years [31]. The use of the PRP is recently an attractive approach for enhancing and accelerating the tenocyte proliferation. It is a minimally invasive and a relatively low-cost therapeutic strategy. Injection of autologous platelets was shown to increase the tissue healing, such as rotator cuff tears [8,9,10]. In contrast, it also reported no significant difference or even worse clinical outcomes [11,12,13,14,15]. One possible reason might be the variability of the patients, such as age, gender, and level of injury. For example, tenocytes from supraspinatus had reduced cell growth and collagen production in female patients older than 65 years [31]. The cell biological characteristics of tenocytes were inferior in elderly patients. Thus, more basic studies are required to figure out the characteristics of the PRP and the variability of the patients. The 3-chamber co-culture device becomes a tailor-made tool for the PRP study.

## 4. Conclusions

In this work, a 3-chamber co-culture device has been designed and developed for the PRP study. Reduction of the usage of primary cells and avoidance of the PRP gelation effect are the objectives of the device development. The tenocytes and the PRP were partitioned and co-cultured under the interlinked medium. Thus, the platelet-derived growth factors could diffuse in the medium and enhance cell proliferation. The results showed that higher platelet number in the PRP could diffuse higher concentrations of the growth factors in the medium and induce higher tenocyte proliferation. The 3-chamber co-culture device was confirmed to be a simple and practical tool for the PRP study. As the efficacy of the PRP therapy is clinically unclear and remains controversial among medical experts, the device allows large volume in vitro studies using clinical samples.
